# Atrial tachycardia misinterpreted as ventricular high‐rate event in a patient with the sick sinus disease and a DDD pacemaker

**DOI:** 10.1002/joa3.12497

**Published:** 2021-01-04

**Authors:** Saurabh Ajit Deshpande, Ameya Udyavar

**Affiliations:** ^1^ Department of Cardiac Electrophysiology Sri Jayadeva Institute of Cardiovascular Sciences and Research Bangalore India; ^2^ Department of Cardiology P D Hinduja Hospital Mumbai India

## Abstract

The device misinterpreted atrial high‐rate episode (AHRE) as ventricular high‐rate episode (VHRE), since consecutive atrial beats fell in the postventricular atrial blanking (PVAB) period. This resulted in the failure of auto mode switch (AMS). This was corrected by decreasing the PVAB and the patient was asymptomatic on follow‐up.

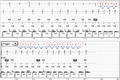

## 1 BACKGROUND

**FIGURE 1:**
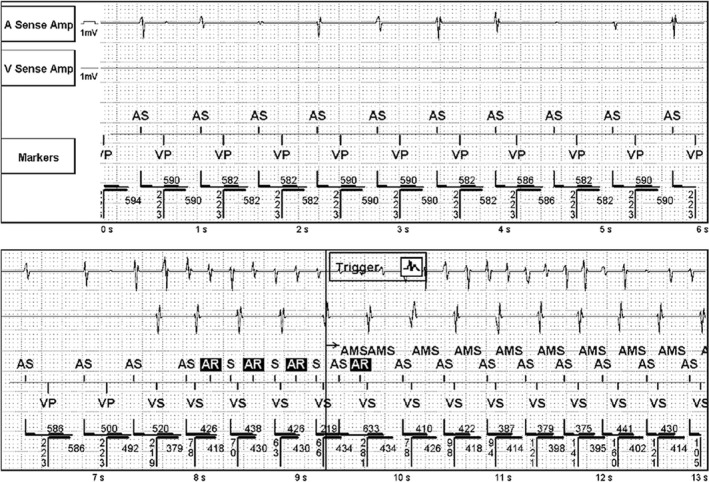
Appropriate mode switch

**FIGURE 2:**
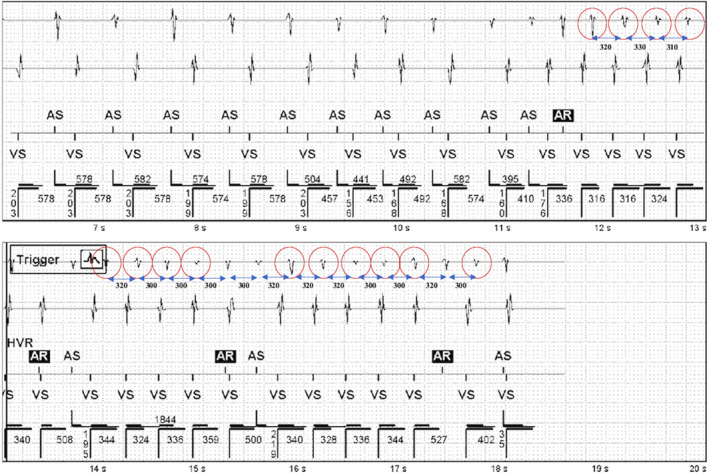
Ventricular High‐Rate Episode (red circles denote the atrial beats which the device failed to annotate; blue bidirectional arrow and accompanying numbers represent the cycle length)

A 66‐year‐old male, known diabetic and hypertensive, with significant sinus pauses and syncope underwent a dual‐chamber DDD pacemaker (SJM Accent DR RF 2212 with base rate of 60 bpm and maximum tracking rate of 125 bpm) implantation for sick sinus syndrome. The ventricular and atrial leads were positioned at the right ventricular septum and right atrial appendage, respectively. His biventricular function was normal. He was asymptomatic on follow‐up. The follow‐up interrogation showed a good battery life (2.93 V, 5 years); normal lead function [Atrial – Sensed *P* = >5 mV, Threshold = 0.625 V@0.4 ms, Impendence = 360Ω; Ventricular – Sensed R = >12 mV, Threshold = 0.75 V@0.4 ms, Impedance = 450Ω] with nominal sensitivities (Atrial – 0.5 mV; Ventricular – 2.0 mV); PAV = 250 ms & SAV = 225 ms; PVARP = 275 ms and PVAB = 150 ms It showed 17 episodes of auto mode switch (AMS) and five ventricular high‐rate episodes (VHREs) [Mode switch settings: Trigger 180 bpm; DDD→DDIR]. The interrogation of the episodes showed that the VHREs were atrial high‐rate episodes (AHREs) in which there was a failure of the mode switch. The interrogation of the episodes of AMS (Figure 1) and VHRE (Figure 2) has been shown in the figures. What can be the reason for the failure of the pacemaker to annotate these episodes as VHREs instead of atrial fibrillation? How can it be corrected?

## 2 DISCUSSION

Dual‐chamber pacemakers detect atrial and ventricular high‐rate episodes (VHREs). Most common arrhythmia in patients with sick sinus syndrome is subclinical atrial fibrillation. When atrial fibrillation is detected, the pacemaker causes mode switch to a nontracking mode, which is termed as an auto mode switch (AMS). There can be situations where the mode switch has failed to work.

As it is seen in Figure 1, the patient had a supraventricular tachycardia during these episodes as there were more atrial electrograms (EGMs) than ventricular EGMs in the EGM sensing channels. The marker channels have annotated all the atrial beats and hence the pacemaker has had an appropriate mode switch to DDI mode (nontracking mode). It should be noted that the smallest of the “a” waves were detected during this episode, confirming a good atrial lead sensitivity.

As against, in Figure 2, the similar supraventricular tachycardia is seen in which a few of the atrial beats were failed to be annotated as either A‐sense (As) or A‐refractory (Ar) in the marker channel (all such beats are circled in Figure 2). So, the pacemaker considered the presence of more ventricular events than atrial events, labeling it to be a VHRE. The reason for this is that all the atrial beats which were not annotated fell in the postventricular atrial blanking (PVAB) period. AMS switches pacemaker from DDDR to DDIR mode (ventricular rate to AMS base rate) only when the atrial rate surpasses the atrial tachycardia detection rate. This episode was not detected as atrial high‐rate episodes (AHREs) and hence there was a failure to cause mode switch. Similar scenario may be observed in the situations where a short RP tachycardia (where the retrograde p falls in PVAB) can be misinterpreted as VHRE.

Over the years, the pacemakers have improved programing algorithms to detect and prevent tracking of supraventricular events. AMS algorithms are specific for preventing increased ventricular rate because of atrial tracking in case of AHRE. AMS is influenced by characteristics of arrhythmia, programed parameters (such as PVAB), and characteristic of individual AMS algorithm (ie, accuracy, onset, AMS response, and resynchronization).^1^

Undersensing is a common problem which can cause AMS failure because of the failure of detection of the small atrial event or because of the pacemaker being less sensitive to detect smaller atrial events. AMS failure may occur when the atrial event consistently falls in PVAB, as seen with atrial flutter.^2^ In our case, the smallest of atrial deflections out of PVAB were detected, so there was no undersensing. The cause of underdetection was the atrial event falling into PVAB, which was rectified with a decrease of PVAB to 80 msec. Reducing the PVAB further is not advisable in every case as this might pick up the QRS as atrial activity resulting in a “false” mode switch.

## 3 LEARNING OBJECTIVES

In patients with DDD pacemakers, detailed interrogation of VHREs and analysis of the EGMs and their annotations should be carried out to prevent a supraventricular event from being detected as a ventricular event. This can have clinical implications for the patient in means of treatment and prognosis. Careful adjusting of the PVAB can help prevent this problem.

## CONFLICT OF INTEREST

Authors declare no conflict of Interests for this article.
